# Does Lithium Deserve a Place in the Treatment Against COVID-19? A Preliminary Observational Study in Six Patients, Case Report

**DOI:** 10.3389/fphar.2020.557629

**Published:** 2020-08-27

**Authors:** Carlos Spuch, Marta López-García, Tania Rivera-Baltanás, Daniela Rodrígues-Amorím, José M. Olivares

**Affiliations:** ^1^ Translational Neuroscience Research Group, Galicia Sur Health Research Institute (IISGS), CIBERSAM, Vigo, Spain; ^2^ Department of Psychiatry, Hospital Álvaro Cunqueiro, Translational Neuroscience Research Group, Galicia Sur Health Research Institute (IISGS), CIBERSAM, Vigo, Spain; ^3^ Department of Psychiatry, Hospital Álvaro Cunqueiro, Vigo, Spain; ^4^ Neuroscience Research Area, Galicia Sur Health Research Institute (IISGS), CIBERSAM, Vigo, Spain

**Keywords:** SARS-CoV-2, COVID-19, lithium carbonate, case report, inflammation

## Abstract

Lithium has shown the capacity to: a) inhibit the replication of several types of viruses, some of which are similar to the SARS-CoV-2 virus, b) increase the immune response by reducing lymphopenia, and c) reduce inflammation by preventing or reducing the cytokine storm. In the present study, we have treated six patients with severe COVID-19 infection with lithium carbonate. We found that lithium carbonate significantly reduced plasma reactive C-Protein levels, increased lymphocyte numbers and decreased the neutrophil-lymphocyte ratio, improving both inflammatory activity and the immune response in these patients. We propose that lithium carbonate may deserve a place in the treatment against COVID-19.

## Introduction

Lithium salts belong to a class of medications termed anti-manic agents, or mood stabilizers. It is used in Spain and other countries in the form of lithium carbonate (Plenur^R^), mainly as a mood stabilizer in people diagnosed with bipolar disorder, in order to treat acute episodes of mania (frantic, abnormally excited mood) and prevent recurrences of both mania and depression. It is also used in some cases of resistant depression, and it is the only psychiatric medication (other than clozapine) proven to prevent suicide (Baldessarini et al., 2003). Clinically, lithium has also been used to treat severe psychotic symptoms in some patients with schizophrenia (Leucht et al., 2015) and it has also been prescribed to control irritability and aggressive behaviors in several conditions, such as personality disorders, children with conduct disorders and aggressive subjects with intellectual disability (Tyrer, 1994). Some authors consider that, with the exception of ECT, lithium is the single most effective treatment in Psychiatry (Shorter, 2009).

Apart from this, lithium seems to have other potential clinical uses, and we will focus first on its antiviral properties. There is some evidence that lithium can inhibit the replication of several types of viruses, including some belonging to the coronavirus family. In 1979, Julian Lieb observed that patients with manic depression treated with lithium carbonate showed clinical remission of recurrent herpes simplex virus (HSV) infection (Lieb, 1979; Lieb, 1981). In 1980, Skinner’s group showed that lithium inhibits HSV replication in the kidney cells of a baby hamster (Skinner et al., 1980). More recent evidence corroborates the antiviral effect of lithium against some DNA and RNA type viruses, such as HSV, infectious bronchitis virus, canine parvovirus, porcine parvovirus, pseudorabic herpes virus, porcine reproductive and respiratory syndrome, transmissible gastroenteritis virus, vaccinia virus, human immunodeficiency virus, and feline calicivirus (Harrison et al., 2007; Cui et al., 2015; Wu et al., 2015; Zhai et al., 2019; Zhao et al., 2020).

The anti-viral properties of lithium salts seem to be related to the activity of the lithium ion and its competition with magnesium ions (Bach, 1987). Magnesium acts as a cofactor for enzymes that are needed for the replication of viral proteins and nucleosides. When lithium replaces magnesium, it seems to inactivate the polymerase enzymes. The binding (or association) constant of both lithium and magnesium with adenosine triphosphate (ATP) are of the same order of magnitude, explaining why lithium may be able to interfere with virus replication by disrupting key enzymes of the ATP/ADP balance such as ATPases (Briggs et al., 2016).

Lithium is also able to produce some interesting changes in certain cells of the immune system. Specifically, there is evidence showing that lithium is able to regulate both B and T lymphocytes, i.e., lithium can increase the activity of B lymphocytes and reduce the proportion of circulation-suppressing T cells to cytotoxic T cells (Kibirige et al., 2013). Another recent study showed how lithium may have an anti-apoptotic effect on T-lymphocytes of patients with bipolar disorder (Pietruczuk et al., 2018). Lithium also seems to be able to modulate the expression of many genes within lymphocytes themselves (Anand et al., 2016), implying that its effects on lymphocytes are much more complex than simply altering lymphocyte populations, and leads one to the hypothesis that lithium could act as a metabolic modulator of lymphocytes (Jellusova et al., 2017).

Finally, there is accumulating evidence suggesting that lithium may exert some anti-inflammatory effects, such as suppression of cyclooxygenase-2 expression, inhibition of interleukin (IL)-1β and tumor necrosis factor-α production, and enhancement of IL-2 and IL-10 synthesis (Nassar and Azab, 2014). In summary, lithium is a drug with antiviral properties against several virus types (including some within the coronavirus family), and it has some capacity to regulate the immune system and increase anti-inflammatory events. These findings led us to speculate that, at least from a theoretical point of view, lithium could be a good candidate to treat patients affected by COVID-19.

## Case Description

### Prestudy Evaluation

The latest data (April 2020) on the incidence of COVID-19 in Spain reflect that there are 200,210 people infected and there have been 22,524 fatalities (an incidence around 43 per thousand inhabitants). In Galicia (a region in north-western Spain), COVID-19 incidence rates are lower, i.e., 8,304 cases and 463 deaths (incidence of 3.07 per thousand inhabitants). In the Health Area of Vigo, where most of the population in Galicia is concentrated, 1,633 cases and 108 deaths have been recorded for COVID-19, an incidence of 2.89 per thousand inhabitants. In order to explore some preliminary evidence on lithium’s effectiveness on COVID-19 infection, we compared our database of patients receiving lithium in our catchment area (437 patients) with the registry of patients positive for COVID-19. We found that none of them were infected. This finding does not prove that lithium is effective against COVID-19, but if a number of patients taking lithium were to be infected, then our hypothesis would have been considerably nullified.

The next step in search for evidence for the plausible efficacy of lithium in treating patients with COVID-19 was to prescribe lithium carbonate to some COVID-19-positive patients whom also present psychiatric symptoms, which would justify, at least from a psychiatric viewpoint, the use of the drug. This was assessed independently by two of the authors who are both clinical psychiatrists (MLG and JMO). Finally, we selected six hospitalized patients, all of whom were over 16 years old and had provided written informed consent for the publication of any potentially identifiable images or data included in this article.

Blood lithium levels were measured after 48 h after the initial dose of lithium carbonate was given. We were looking for plasma levels in the lower range of efficacy in the usual treatment of bipolar patients (0.6–0.8 mEq/L), while not allowing, in any case, plasma levels over 1.2 mEq/L to avoid toxicity. If an adjustment in dose was required to reach those levels, a new measurement of plasma levels would be performed 72 h after the first intake of the increased dose. Special consideration was given to any concomitant diuretic and/or antiretroviral treatment the patient could be taking, as those may modify (i.e., increase) the plasma levels of lithium in the blood and QTc, respectively. Finally, we compared the 6 COVID-19-positive patients on regular treatment plus lithium carbonate with 3 patients with COVID-19 on regular treatment.

### Phenotypes of the Patients

Patient 1: 64-year-old male, diabetes mellitus, dyslipaemia, and arthrosis with lumbar discopathy, attending the emergency department showing fever and dry cough, with a blood pressure of 160/90, a heart rate of 85 beats per minute, a SpO2 measure of 94%, and elevated D-Dimer levels. Chest X-ray: Faint opacities in the upper right and lower left lobes were seen, suggesting the presence of infectious and viral processes and atypical germs. Confirmation of COVID-19 was made by RT-PCR using a throat swab. The patient was treated with lopinavir-ritonavir, hydroxychloroquine (for 5 days), and lithium carbonate (starting on day 5, initial dose of 200 mg/12 h and after 48 h increased to 400 mg/12 h). He was also was treated with prednisolone (day 14, 40 mg/8). The patient was discharged after 27 days.

Patient 2: 60-year-old male, the patient has no medical history with relevance to COVID-19, attending the emergency department for fever and dry cough lasting 1 week, with a blood pressure of 115/60, a heart rate of 91 beats per minute, SpO2 98%, and elevated D-Dimer levels. Chest X-ray: Diffuse bilateral interstitial pattern with opacities in the middle third of the right hemithorax and both lung bases was detected, probably related to atypical infectious pneumonia. Confirmation of COVID-19 was made by RT-PCR using a throat swab. He was treated with hydroxychloroquine (for 5 days), and then with lithium carbonate (starting on day 5, 400 mg/d). The patient was discharged after 20 days.

Patient 3: 40-year-old female, with dyslipemia, chronic venous insufficiency, obesity, attending the emergency department for bilateral pneumonia with respiratory failure. Her blood pressure was 107/73, heart rate 79 beats per minute, SpO2 92%, and she had elevated D-Dimer levels. Home treatment: Lorazepam (1 mg), olanzapine (10 mg), risperidone (3 mg), lactitol monohydrate and lactulose. Chest X-ray: Interstitial pattern of right and left basal predominance, with patchy opacities in the middle and upper third of the right hemithorax, suggestive of a viral infectious process. Confirmation of COVID-19 was made by RT-PCR using a throat swab. She was treated with hydroxychloroquine (for 5 days), lopinavir-ritonavir and lithium carbonate (starting on day 2, 400 mg/12 h). The patient was discharged after 11 days.

Patient 4: 54-year-old male, healthy carrier of the hepatitis B virus, attending the emergency department for bilateral pneumonia, fever and with respiratory failure. He had a blood pressure of 91/58, a heart rate of 73 beats per minute, SpO2 88%, and elevated D-Dimer levels. Chest X-ray: Bilateral interstitial pattern with opacities in ground glass, suggesting a viral or atypical infectious process. Confirmation of COVID-19 was made by RT-PCR using a throat swab. He was treated with lithium carbonate (400mg/12h). The patient was discharged after 18 days.

Patient 5: 68-year-old male, chronic obstructive pulmonary disease and benign prostatic hyperplasia, attending the emergency department for increased dyspnoea in the last 48 h, together with cough and dark expectoration. His blood pressure was 112/81, heart rate 82 beats per minute, SpO2 95%, and elevated D-Dimer levels. Chest X-ray: He had a small nodular infiltrate in the upper left lobe suggesting an infectious process. The radiological pattern was indicative of emphysema (two-basal, varicose bronchiectasis in left hemithorax). Confirmation of COVID-19 was made by RT-PCR using a throat swab. He was treated with hydroxychloroquine (for 5 days), risperidone, lopinavir-ritonavir and lithium carbonate (first dose on day 12, 200 mg/12 h and second dose on day 15, with 600 mg/12 h). The patient was discharged 9 days later.

Patient 6: 52-year-old male, the patient has no medical history with relevance to COVID-19, attending the emergency department presenting fever, dry cough and respiratory failure, with a blood pressure of 101/64, a heart rate of 101 beats per minute, SpO2 93%, and elevated D-Dimer levels. Chest X-ray: An endotracheal tube was inserted 3cm from the carina and a right subclavian central venous catheter was positioned. Diffuse bilateral interstitial pattern with ground glass opacities in middle and lower right lobe was observed, which was indicative of an atypical viral infection. Confirmation of COVID-19 was made by RT-PCR using a throat swab. The patient was treated with lopinavir-ritonavir, hydroxychloroquine (during the first 5 days), ceftriaxone, azithromycin, corticoids (day 7), and lithium carbonate (400 mg/12 h) since day 7. The patient remains in the intensive care unit.

Control 1: 57-year-old male with obesity, attending the emergency department for fever that was present for 10 days, as well as a cough and respiratory failure. His blood pressure was 121/78, heart rate 110 beats per minute, SpO2 94-95%, and he had elevated D-Dimer levels. Chest X-ray: A bilateral alveolar reticulum interstitial pattern of peripheral predominance and right base was observed which was suggestive of an atypical infection. Confirmation of COVID-19 was made by RT-PCR using a throat swab. The patient was treated with hydroxychloroquine and lopinavir-ritonavir, and they remain in the hospital.

Control 2: 66-year-old male with ischemic dilated cardiomyopathy, whom had a heart transplant in 2011 and carries a pacemaker, attending the emergency department for persistent fever, dry cough and with respiratory failure. His blood pressure was 143/67, heart rate 70 beats per minute, SpO2 95%, and he had elevated D-Dimer levels. Chest X-ray: We found a minimal reticular interstitial pattern of basal predominance. Confirmation of COVID-19 was made by RT-PCR using a throat swab. The patient was treated with hydroxychloroquine, prednisone, lopinavir-ritonavir, vancomycin, and tacrolimus, and he remains in the intensive care unit.

Control 3: 72-year-old male with diabetes mellitus, chronic ischemic cardiopathy and dyslipaemia, attending the emergency department for cough and fever during the last 15 days without improvement and accompanied by diarrhea. He also had partial respiratory failure. His blood pressure score was 154/81, and he had a heart rate of 80 beats per minute, SpO2 87%, and elevated D-Dimer levels. Chest X-Ray: A bilateral alveolar reticulum interstitial pattern of peripheral predominance and right base was observed, indicating an atypical infectious process. Confirmation of COVID-19 was made by RT-PCR using a throat swab, as well as testing positive for pneumococcus. He was treated with hydroxychloroquine, prednisone, lopinavir-ritonavir, vancomycin, and tacrolimus. The patient remains in the intensive care unit.

## Statistical Analyses

The effects of lithium treatment between dependent variables were evaluated with regression analyses using GraphPad Prism 7.0 software. Multiple linear regression is one of the most important statistical methods used to model the linear relationship between a dependent variable and one or more independent variables to fit an exponential decay curve. We calculated a nonlinear regression with one phase exponential decay.

## Material and Methods

### Laboratory Parameters

Blood samples were collected and sent to the laboratory for analysis. CRP levels were measured by nephelometry. Total number of white blood cells, neutrophils, and lymphocytes were obtained using an automated blood cell counter. NLR was calculated as the ratio neutrophil/lymphocyte count. PLR was calculated as the ratio platelet/lymphocyte count. Both ratios were obtained from the same automated blood sample at the laboratory.

**Figure 1:**
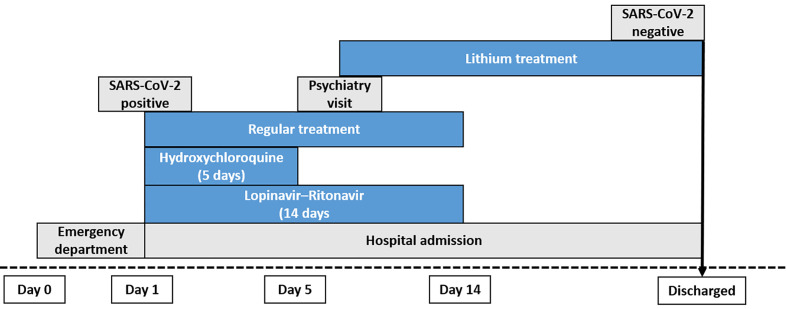
Diagram of the workflow and treatment followed for each patient treated with lithium carbonate. All of the lithium-treated and control patients, with the exception of patient 4, were treated with regular COVID-19 therapy (hydroxychloroquine for 5 days and lopinavir-ritonavir for 14 days).

Patients with symptoms of COVID-19 enter the hospital through the emergency department. They are diagnosed by RT-PCR as being positive for SARS-CoV-2. Patients are admitted to the hospital and given the regular treatment (hydroxychloroquine for 5 days and lopinavir-ritonavir for 14 days) (Sinha and Balayla, 2020; Stower, 2020). All nine COVID-19 patients selected for this study were provided regular treatment to ameliorate the SARS-CoV-2 infection, eight of whom received hydroxychloroquine for 5 days and lopinavir-ritonavir combination therapy. Internal medicine doctors decided not to treat patient 4 with hydroxychloroquine and/or lopinavir-ritonavir. Six of these patients were treated with lithium carbonate after psychiatric evaluation by two different psychiatrists who considered they could benefit from receiving the treatment. Patients 1 and 5 received an initial dose of 200 mg/12 h, and after plasma lithium levels were measured (after 48 h from first dose) the dose was increased to 400 mg/12 h for patient 1 and 600 mg (200 mg-0-400 mg) for patient 5. The rest of the patients received 400 mg/12 during 48 h and after plasma lithium levels were measured (0.6–1.2 mE/L) they remained on the same dose. In the case of patient 4, physicians did not consider hydroxychloroquine and lopinavir-ritonavir treatment because he had been suffering from COVID-19 symptoms for more than 12 days, so he only received lithium carbonate (400 mg/12 h) (**Figure 1**).

## Results

**Figure 2:**
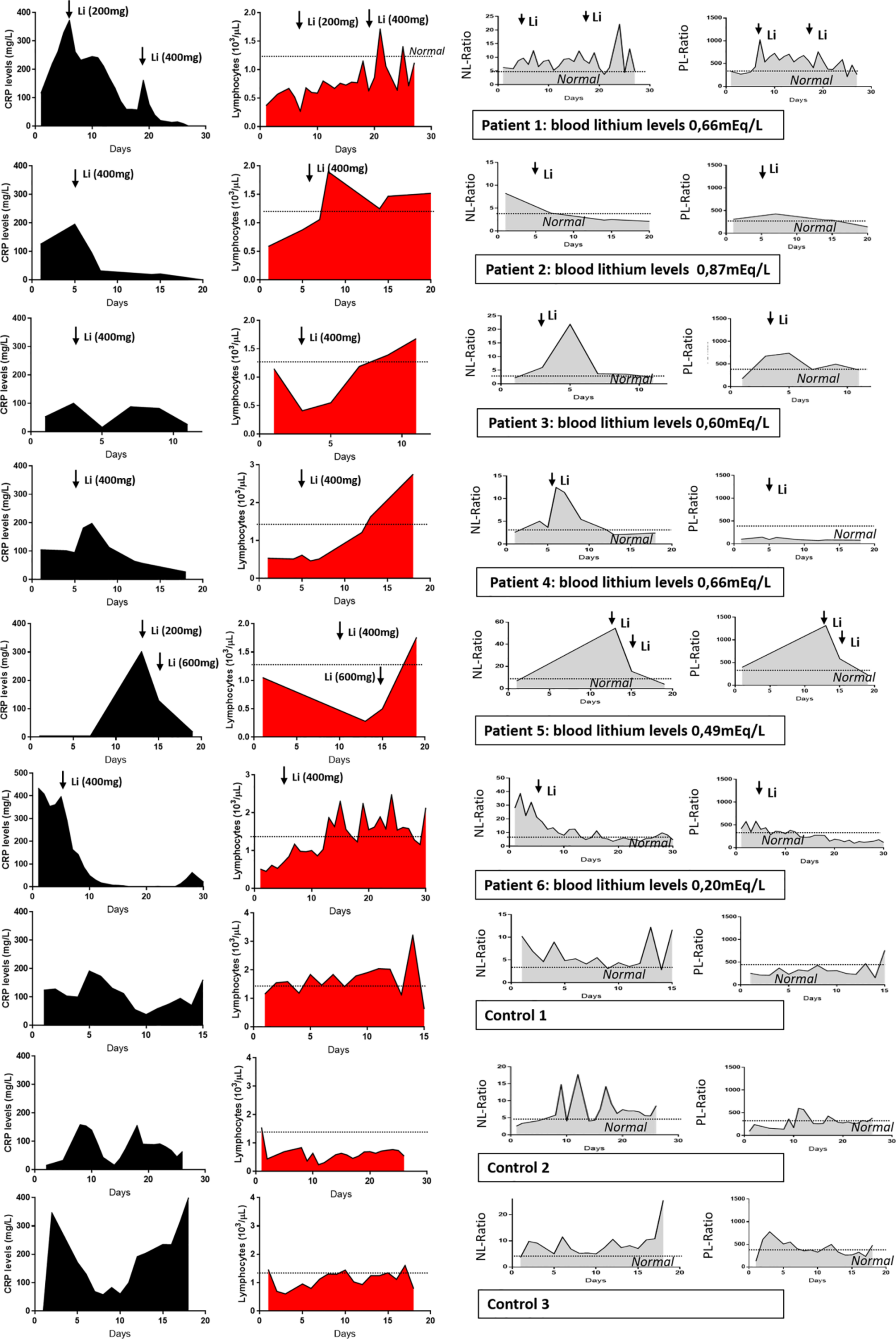
Effect of lithium therapy on individual COVID-19 patients. C-reactive protein blood levels, number of lymphocytes and neutrophil-lymphocyte ratio and Platelet-lymphocyte ratio in six COVID-19 patients treated with lithium carbonate (plus regular therapy) and three control patients with regular therapy only.

All patients that were treated with lithium carbonate (**Figure 2**) had improvements in their CRP levels. Patients 1 and 5, who had their dose of lithium carbonate increased, experienced greater improvements after the second dose. Similarly, when we analyzed the number of lymphocytes, we saw that lithium carbonate treatment improved the levels of lymphocytes until they returned to normal levels. In the case of patient 4, who was only treated with lithium carbonate, the drop in CRP levels and the increase in lymphocytes took 3 days.

We then calculated the neutrophil-lymphocyte ratio (NLR) levels as a marker of inflammation, as it is associated with heightened levels of pro-inflammatory cytokines (Chen et al., 2015). All six patients treated with lithium carbonate showed a sharp reduction in the NLR. In patient 3, this ratio decreased two days after the initial dose, probably because plasma levels were not yet in the therapeutic range.

We have also calculated the platelet-lymphocyte ratio which has been described, together with the NLR, to have prognostic value in severe cases (Qu et al., 2020). This parameter was also improved in patients treated with lithium carbonate, in a similar manner as the NLR ratio.

When comparing these data with that of the three controls, one should consider that the lithium carbonate treatment was given to patients with a higher severity of infection than controls. However, in the control group, despite being treated with hydroxychloroquine and lopinavir-ritonavir, there was a clear oscillation in the inflammatory parameters and in the lymphocyte count, as the infection progressed, and inflammatory waves can be seen increasing in height and amplitude with time.

**Figure 3:**
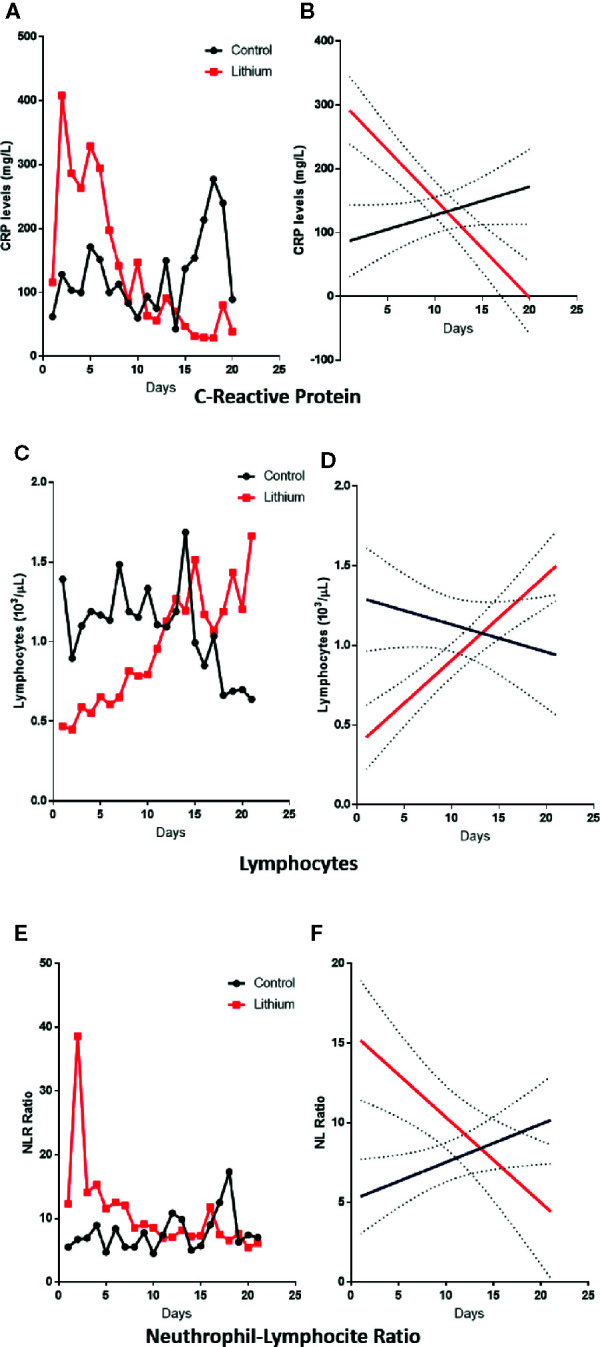
Inflammatory changes induced by lithium. Left panels represent the average behavior of CRP **(A)**, lymphocyte **(C)** and NL-ratio **(E)** measurements for each day. Results show that patients treated with lithium had a more severe infection than controls, and lithium treatment considerably improved all these parameters. Right panels show linear regression analysis, where a clear different pattern among groups is observed (*p*<0.05) with different slopes for CRP **(B)**, lymphocyte **(D)** and NL-ratio **(F)**.

**Figure 3** shows the average of CRP, number of lymphocytes and NLR measurements for both groups. Interestingly, treatment with lithium carbonate improved all three parameters, which were unchanged in the control group. Even though the number of patients included in this study was small, linear regression analyses revealed that the behavior of both curves (patients vs. controls) were completely, and statistically significantly different, favoring the patients on lithium carbonate treatment.

There were no relevant side effects related to lithium carbonate. We did not use any treatment satisfaction questionnaire for medication; however, patients were very gratified. All six patients on lithium survived, one of the three controls died.

## Discussion

We have described here, for the first time, a rationale proposal to treat COVID-19-positive patients with lithium carbonate. We have presented data from a group of six COVID-19 patients on standard treatment plus lithium carbonate vs. three control cases who only received standard treatment.

CRP is a group of proteins that increase in response to inflammatory cytokines, mainly in the early stages of infection (Ling, 2020; Tan et al., 2020; Terpos et al., 2020). All six patients in our experimental group had very high CRP levels and were in a more severe condition than those in the control group. When treated with lithium carbonate, the patients’ CRP levels improved considerably before finally reaching normal levels.

On the other hand, severely affected COVID-19 patients also develop severe lymphopenia (Terpos et al., 2020). During hospitalization, non-survivors demonstrated a more significant deterioration in lymphopenia compared with those who survived (Wang et al., 2020). Patients on lithium carbonate were able to quickly increase the number of lymphocytes and finally achieved normal lymphocyte values.

Another parameter that suggests a worsened evolution of the infection is the NLR, which is significantly associated with elevated cytokine levels and a poorer disease prognosis (Lagunas-Rangel, 2020; Liu et al., 2020). In patients treated with lithium carbonate, we observed that the NLR decreased after treatment, and in those patients where it was necessary to increase the lithium dose, a greater improvement was seen until they normalized completely.

Platelet/lymphocyte ratio (PLR) has been described recently, together with the NLR, as a prognostic factor for the severity of COVID-19 infection (Terpos et al., 2020). In our case series we found that, along with the NLR, PLR was also completely normalized by lithium treatment. These data suggest that lithium treatment reduces the inflammatory state of patients with COVID-19, by lowering CRP levels and improving the NLR and PLR. In addition, another positive effect of lithium carbonate is that it seems to improve their immunological effects by increasing lymphocyte levels. All patients treated with lithium carbonate improved their clinical status and were discharged (except patient 6, who remains in the ICU but with no more need for ventilation).

One question that may arise is whether raising lymphocyte levels affects IL-6 or ferritin by other mechanisms. However, in our series of patients we found no evidence that lithium carbonate modified IL-6 or ferritin levels. A recent study showed that in hospitalized adult patients with severe COVID-19, no benefit was observed with lopinavir–ritonavir treatment beyond standard care (Cao et al., 2020). In our case we have shown that lithium carbonate treatment could be effective and with fewer side-effects. We do not know if lithium carbonate is able to reduce the viral load of SARS-CoV-2, which still needs to be tested, but here we have provided evidence that all patients treated with lithium were RT-PCR test negative at discharge. COVID-19 is a disease with no single effective treatment to date. Until the first vaccines are available, there is a need for an effective and preferably inexpensive treatment that can be administered worldwide. Lithium is a drug that has been used by doctors for the last 70 years, and it has been shown to have antiviral properties against different RNA and DNA viruses. We hope that we will be able to elucidate its promising antiviral actions against COVID-19 in future research. It has also been shown elsewhere that lithium may influence inflammation and lymphocyte count. As we now know, lymphocyte numbers are largely decreased by COVID-19 in severely affected patients, and interestingly, we have seen how this is reversed in all six patients treated with lithium.

In conclusion, treatment with lithium carbonate may open a new therapeutic possibility to treat COVID-19-positive patients. We are aware of the severe limitations of this study such as the small number of cases and the lack of randomization. However, we still believe that these preliminary results should be available to the scientific community, encouraging other colleagues to conduct new studies that may lead to the evaluation of lithium as a potential treatment for severe cases of COVID-19 infection.

We have also asked for permission to start a clinical trial to overcome these limitations (EudraCT number 2020-002008-37).

## Data Availability Statement

The raw data supporting the conclusions of this article will be made available by the authors, without undue reservation.

## Ethics Statement

The studies involving human participants were reviewed and approved by Comité de Ética de Investigación con Medicamentos de Galicia. 2020–238. The patients/participants provided their written informed consent to participate in this study.

## Author Contributions

CS, TR-B, and DR-A analyzed and studied all the cases. ML-G and JO performed the patient recruitment and treatments. CS and JO wrote the manuscript.

## Funding

This research was financially backed by the Foundation for Science and Technology (FCT, Fundação para a Ciência e Tecnologia) within the framework of grant SFRH/BD/135623/2018 awarded to DR-A. Our research was further supported by the Carlos III Health Institute (ISCIII, Instituto Carlos III) through grant P16/00405, and the Ministry of Health, Equality, and Social Policy (Ministerio de Sanidad, Servicios Sociales e Igualdad) – Government Delegation for the National Plan on Drugs (Delegación del Gobierno para el Plan Nacional sobre Drogas) through grant number 2017I054 awarded to JO. Finally, this work was also supported by the Consolidation and structure programme of competitive research units (Consolidación y estructuración de unidades de investigación competitivas); through grant number IN607B 2018/17.

## Conflict of Interest

The authors declare that the research was conducted in the absence of any commercial or financial relationships that could be construed as a potential conflict of interest.
